# Targeting MDM4 Splicing in Cancers

**DOI:** 10.3390/genes8020082

**Published:** 2017-02-20

**Authors:** Boris Bardot, Franck Toledo

**Affiliations:** Génétique de la Suppression Tumorale, Equipe Labellisée Ligue, Institut Curie, Centre de Recherche, Sorbonne Universités, UPMC Univ Paris 06, CNRS UMR 3244, PSL Research University, 26 rue d’Ulm, 75248 Paris CEDEX 05, France

**Keywords:** MDM4, MDM2, p53, alternative splicing, isoform

## Abstract

MDM4, an essential negative regulator of the P53 tumor suppressor, is frequently overexpressed in cancer cells that harbor a wild-type P53. By a mechanism based on alternative splicing, the *MDM4* gene generates two mutually exclusive isoforms: *MDM4-FL*, which encodes the full-length MDM4 protein, and a shorter splice variant called *MDM4-S*. Previous results suggested that the *MDM4-S* isoform could be an important driver of tumor development. In this short review, we discuss a recent set of data indicating that *MDM4-S* is more likely a passenger isoform during tumorigenesis and that targeting *MDM4* splicing to prevent MDM4-FL protein expression appears as a promising strategy to reactivate p53 in cancer cells. The benefits and risks associated with this strategy are also discussed.

## 1. Alternative Splicing and Cancers: Generalities

Alternative splicing plays a major role in generating protein diversity, by including or excluding specific exons [1]. In addition, by promoting intron retention, exon skipping or inclusion of so-called “poison exons”, it may lead to the expression of isoforms with premature stop codons. As the large majority of these transcripts are sequestered and degraded in the nucleus [2,3] or encode for very unstable proteins [4], alternative splicing is thus also a fundamental process to regulate gene expression. In humans, most multi-exonic genes are alternatively spliced and a tremendous amount of work remains to evaluate the biological impact of the different isoforms encoded by a single gene in physiological or pathological conditions.

Aberrant splicing is a common feature of cancer cells [5]. Pathways frequently deregulated in cancer often play important roles in promoting aberrant splicing which in turn could affect tumor development [6]. For instance, it has recently been shown that the regulation of the core pre-mRNA splicing machinery by the *c-Myc* oncogene is an essential step in lymphomagenesis [7]. Additional evidence that aberrant splicing is important in promoting tumor development came from the observation that recurrent somatic mutations of genes encoding core subunits of the spliceosome, a complex of small noncoding RNAs and various associated proteins catalyzing splicing reactions [8], have been identified in several types of cancers [9]. However, whether the aberrant expression of a given splicing isoform observed in a given cancer contributes to tumor progression (driver isoform) or is simply its consequence (passenger isoform) must be carefully examined. Several cases of such driver isoforms have been described [6]. One of the earliest and most well-known examples is the Bcl-xL isoform encoded by the *Bcl-x* gene [10]. *Bcl-x* encodes two alternatively spliced isoforms with opposite activities. Whereas the short isoform (Bcl-xS) has pro-apoptotic activities, the long one (Bcl-xL) has an anti-apoptotic effect and is highly expressed in a wide range of tumors [11,12]. The specific inhibition of Bcl-xL expression with the antisense oligonucleotide targeting the alternative splice site has been shown to promote apoptosis in hepatocellular carcinoma cells [11]. 

Accumulating data suggest that targeting RNA splicing in tumor cells might be exploited for therapeutic purposes. Indeed, recent results suggest that the spliceosome might represent the Achilles heel of tumor cells. For instance, Myc-driven cancer cells replicate rapidly and may depend on their ability to sustain an increased RNA and protein production, making them particularly vulnerable to any splicing stress. Accordingly, it has been demonstrated that, in contrast to normal cells, partial inhibition of the spliceosome impairs survival of Myc-dependent cancer cells [13]. However, even if global interference of the splicing machinery might be the sand in the gears that block the tumor, it might also have deleterious side effects. For this reason, much effort is still needed to identify the specific cancer-promoting splicing isoforms that are essential to maintain the tumor phenotype, because their specific elimination would be a potent therapeutic strategy. In the following section, we will discuss a recent set of data suggesting that targeting *Mdm4* splicing might be a promising strategy against cancer cells.

## 2. Targeting MDM4 Splicing: A Promising Anti-Cancer Therapy

The MDM4 protein (also known as MDMX) was discovered 20 years ago as a p53 binding protein that shows high structural similarity to MDM2 [14]. Both MDM2 and MDM4 are essential negative regulators of the p53 tumor suppressor and are frequently overexpressed in a great number of tumors that harbor wild-type p53. These properties made them attractive targets to reactivate p53 in tumors [15,16]. Accordingly, several in vivo studies underlined the addiction of various tumor cells to MDM4 [17,18,19,20,21,22,23]. 

By a mechanism based on the inclusion or the skipping of exon 6, the *MDM4* gene generates two alternative transcripts. The one containing exon 6 encodes the full-length MDM4 protein (MDM4-FL), whereas the skipping of exon 6 results in a frame-shift and a premature stop codon. This second alternative transcript encodes a short carboxy-truncated MDM4 protein (MDM4-S) containing the p53 binding domain and a few amino acids of an unrelated sequence due to the frame-shift [24]. Overexpression experiments initially led to the proposal that the MDM4-S protein would be a stronger p53 inhibitor than MDM4-FL [24,25]. This hypothesis was supported by data suggesting that MDM4-S is more efficiently localized into the nucleus and exhibits a higher affinity to p53 than MDM4-FL, and that MDM4-S lacks an auto-inhibitory sequence present in MDM4-FL [24,25,26]. However, a recent set of data lead us to believe that the main effect of exon 6 skipping is to negatively regulate the expression of MDM4-FL. First, due to the in-frame insertion of a premature stop codon, the transcript lacking exon 6 could be the target of non-sense-mediated decay machinery [27,28]. Moreover, we recently showed that mice engineered for an obligatory Mdm4 exon 6 skipping exhibit increased p53 activity concomitant to Mdm4-FL decrease [29]. Interestingly, the mutant allele (*Mdm4^∆E6^*) also leads to increased Mdm4-S mRNA levels, a situation that has been observed in several cancers and is correlated with a bad prognosis [30,31,32,33]. This allowed us to compare Mdm4-FL and Mdm4-S protein expression in heterozygous *Mdm4^+/∆E6^* mice. Even in a context where the mRNA levels for the Mdm4-S isoform were superior to the mRNA levels for the Mdm4-FL isoform, Mdm4-FL was much more abundant than Mdm4-S at the protein level. Indeed, the Mdm4-S protein was barely detectable, suggesting the existence of post-transcriptional mechanisms that negatively regulate Mdm4-S translation and/or stability. Accordingly, we could significantly increase Mdm4-S protein levels upon proteasome inhibition [29]. 

Because the main effect of the skipping of *MDM4* exon 6 is not the synthesis of the MDM4-S protein, but rather a decrease in MDM4-FL expression, it appeared that promoting *MDM4* exon 6 skipping could be a safe and specific way to decrease MDM4 protein levels in order to reactivate p53 in tumors. Accordingly, it has recently been shown that antisense oligonucleotide-mediated skipping of exon 6 decreased MDM4 abundance and reduced the growth of various human tumor cells [23]. Moreover, because MDM4 has also p53-independent oncogenic activities [20,34,35,36], targeting *MDM4* splicing to prevent MDM4-FL expression in tumor cells may also present an important advantage over other strategies that are only aimed at preventing MDM4/p53 interaction. 

## 3. Targeting MDM4 Splicing: Anticipated Consequences

Even if targeting *MDM4* splicing might be a promising anti-cancer strategy, two important issues must be anticipated and addressed. The first issue concerns the toxicity due to increased p53 activity upon MDM4-FL inhibition in normal tissues. Genetic studies in mice suggested that keeping p53 in check is critical, but that MDM4 inhibition, unlike MDM2 inhibition, might be well tolerated in adult animals [19,37,38,39,40,41]. Nevertheless, this issue must be very carefully examined because the hematopoietic tissue is very sensitive to MDM4 levels and unchecked p53 activity in this tissue may lead to severe bone marrow failure [42]. Whether or not this new targeting strategy is actionable will have to be rigorously tested in preclinical studies using appropriate mouse models. As for all therapeutic strategies, the therapeutic index will indeed determine the full potential of this approach. The second issue concerns the possible acquired resistance to treatment. In tumors, at least two possible escapers could be selected for. First, this therapeutic strategy could favor the emergence of cells generating an abnormal splicing event between MDM4 exons 5 and 8. This splicing event would generate an isoform with a restored open reading frame that would encode a MDM4 protein with an internal deletion but presumably having all the important domains necessary for its oncogenic activities. Such a splicing event has never been described in normal conditions but might arise from a secondary intronic mutation in a tumor cell. A comparable situation is at the origin of acquired resistance to vemurafenib, a very potent BRAF inhibitor, in melanoma treatment [43,44]. Secondly and maybe more likely, it might also select for tumor cells with a mutant p53. Indeed, such a mechanism of acquired resistance was recently identified in patients treated with MDM2 antagonists to reactivate wild-type p53 in tumors [45]. Interestingly, reduced MDM4-FL expression due to alternative *MDM4* splicing often correlates with tumors that express a mutant p53 [46]. Even if an increase in MDM4-S mRNAs might simply result from alterations in the splicing regulatory machinery that frequently occur during tumor progression, this observation raises the intriguing possibility that reducing MDM4-FL expression might confer a selective advantage to tumor cells expressing a mutant p53. Furthermore, a few reports suggested that MDM4 might also have p53-independent tumor-suppressive functions [47,48,49,50]. Thus, it will be important to evaluate the risk of a further decrease in MDM4-FL expression in tumors with mutated p53. The output will depend on the balance between the p53-independent oncogenic and the p53-independent tumor-suppressing activities of MDM4. In melanoma cells, MDM4 inhibition causes a cell-cycle arrest, which could not be rescued upon simultaneous inactivation of p53 [21]. This observation suggests that, at least for melanoma patients, MDM4 inhibition by forced alternative splicing would be beneficial.

## 4. Regulation of *MDM4* Splicing

In melanoma cells where MDM4 protein is often overexpressed, there is no correlation between MDM4 protein levels and total *MDM4* mRNA levels, suggesting that MDM4-FL is mainly regulated at a post-transcriptional level [21]. Accordingly, it has been recently proposed that the mechanism that underlies MDM4 upregulation in different cancer cells depends on a specific alternative splicing switch promoting exon 6 inclusion [23]. 

The identification of factors controlling *MDM4* splicing is the object of intense research. Notably, it has been shown that *MDM4* exon 6 inclusion depends in part of the activity of PRMT5 and SRSF3 splicing regulators. Indeed, either PRMT5 or SRSF3 inactivation leads to *MDM4* exon 6 skipping, loss of MDM4-FL and activation of p53 [7,23,27]. SRSF3 is known to have oncogenic activities and PRMT5 is upregulated following c-Myc activation and contributes to preventing anti-proliferative and pro-apoptotic activities in c-Myc–induced B lymphoma [7,51]. These observations are consistent with the fact that tumor cells expressing a wild-type p53 have a selective advantage to increase the MDM4-FL protein level by promoting *MDM4* exon 6 inclusion. On the other hand, it has been shown that DNA damage induces a splicing switch from *MDM4-FL* to *MDM4-S* mRNA that might contribute to a lower MDM4-FL protein level and thereby activate p53 [52]. Additional works will be required to determine if this switch relies on a DNA damage–induced inhibition of the SRSF3 or PRMT5 splicing regulators.

As a concluding remark, beyond MDM4, many reports of alternative p53 and MDM2 transcripts have accumulated over the last 30 years. Thus, carefully evaluating the roles of the many isoforms of such critical players in the p53 pathway might also lead to the identification of additional potent anti-cancer strategies to be used alone or in combination for the benefit of patients. 
